# Impacts of Clinical Pharmacist Intervention on the Secondary Prevention of Coronary Heart Disease: A Randomized Controlled Clinical Study

**DOI:** 10.3389/fphar.2019.01112

**Published:** 2019-10-08

**Authors:** Huimin Xu, Jie Zou, Xiaoli Ye, Jiayun Han, Lan Gao, Shunbin Luo, Jingling Wang, Chunyan Huang, Xiaofeng Yan, Haibin Dai

**Affiliations:** ^1^Department of Pharmacy, Second Affiliated Hospital, Zhejiang University School of Medicine, Hangzhou, China; ^2^Department of Pharmacy, the 117th Hospital of PLA, Hangzhou, Chin; ^3^Department of Pharmacy, Hangzhou First People’s Hospital, Hangzhou, China; ^4^Department of Pharmacy, Zhejiang Haining People’s Hospital, Jiaxing, China; ^5^Department of Pharmacy, Ganzhou District Zhangye People’s Hospital, Zhangye, China; ^6^Department of Pharmacy, Lishui City People’s Hospital, Lishui, China; ^7^Department of Pharmacy, Ningbo Yinzhou No. 2 Hospital, Ningbo, China; ^8^Department of Pharmacy, The Third Affiliated Hospital of Wenzhou Medical University, Wenzhou, China

**Keywords:** coronary heart disease, coronary artery disease, pharmacist, cardiovascular events, outcome assessment

## Abstract

Coronary heart disease (CHD) is one of the leading causes of morbidity and mortality worldwide, and more efforts should be made to reduce the risk of cardiovascular events. This study aimed to investigate the impact of clinical pharmacist intervention on the prognosis of acute coronary syndrome (ACS) in Chinese patients with CHD. Two hundred and forty patients who had ACS were recruited. Participants were randomly assigned to the intervention group (*n* = 120) or the control group (*n* = 120). The intervention group received a medication assessment and education by the clinical pharmacist at discharge and telephone follow-ups at 1 week and 1 and 3 months after discharge. The control group received usual care. The primary outcomes of this study were the proportion of patients who had major adverse cardiovascular events (MACEs), including mortality, nonfatal myocardial infarction (MI), stroke, and unplanned cardiac-related rehospitalizations within 6 and 12 months after hospital discharge. Secondary outcome was self-reported medication adherence to evidence-based medications for CHD (antiplatelets, statins, β-blockers, and angiotensin-converting enzyme inhibitors or angiotensin receptor blockers). Of 240 enrolled patients, 238 (98.3%) completed 6-month follow-up, and 235 (97.9%) completed 12-month follow-up. There were no significant differences between intervention and control groups in the percentages of patients who incurred MACEs within the 6-month follow-up (3.3% vs 7.6%, respectively, *P* = 0.145) or 12-month follow-up (10.9% vs 12.1%, respectively, *P* = 0.783). Significant improvements were found in the prescribing rates of β-blockers and all four classes of medications at discharge in the intervention group compared with the control group (*P* = 0.001 and *P* = 0.009, respectively). There was no significant difference between the intervention and control groups in the use of all four classes of medications at the 6-month follow-up (48.3% vs 45.8%, respectively, *P* = 0.691) and 12-month follow-up (47.9% vs 46.6%, respectively, *P* = 0.836). The use of β-blockers was nonsignificantly higher in the intervention group than in the control group at the 6-month follow-up (74.2% vs. 64.4%, *P* = 0.103) and 12-month follow-up (74.8% vs 63.8%, *P* = 0.068). Clinical pharmacist intervention had no significant effects on reduction in cardiovascular events among patients with CHD. Further studies with larger sample sizes and longer time frames for both intervention and follow-up are needed to validate the role of the clinical pharmacist in the morbidity and mortality of CHD.

**Clinical Trial Registration:** chictr.org.cn, identifier ChiCTR-IOR-16007716.

## Introduction

Coronary heart disease (CHD) is still one of the leading causes of morbidity and mortality worldwide (GBD 2017 DALYs and HALE Collaborators, 2018). In China, according to data from the 2016 Global Burden of Disease Study, the age-standardized prevalence rate of ischemic heart disease increased by 19.1% from 1990 to 2016 (Liu et al., 2019). Secondary prevention is important to decrease the rates of cardiovascular events after the first acute coronary syndrome (ACS). The use of evidence-based medications such as antiplatelets, statins, β-blockers, and angiotensin-converting enzyme (ACE) inhibitors or angiotensin receptor blockers (ARBs) has been reported to reduce the risk of recurrent nonfatal and fatal disease in patients with CHD (Hamm et al., 2011; Smith et al., 2011). However, when patients with CHD leave the hospital and return to the community, they may have poor medication adherence, which can increase the likelihood of cardiovascular events and mortality (Ho et al., 2006; Gehi et al., 2007; Chowdhury et al., 2013; Castaneda-Amado et al., 2017). Therefore, it is crucial that more efforts should be made to improve medication adherence to improve the prognosis of CHD.

Multimodal individualized interventions performed by physicians or nurses have been proven effective in reducing controllable risk factors and in improving medical therapy and patient outcomes for cardiovascular diseases (CVDs) (Wood et al., 2008; Jorstad et al., 2013; Du et al., 2016). Pharmacists can also provide medication and disease management for patients with CVD (Santschi et al., 2011; Cai et al., 2013; Omboni and Caserini, 2018; Doggrell, 2019). Positive effects of pharmacist care on medication adherence and cholesterol control have been shown in patients with CHD (Faulkner et al., 2000; Straka et al., 2005; Ho et al., 2014). However, pharmacists’ impacts on the mortality and morbidity of CHD remain uncertain. In this study, clinical pharmacists provided additional medication assessment and patient education at discharge and intensive follow-ups after discharge on medication adherence and risk factor control for CHD patients. We explored the impacts of pharmacist care on the mortality and morbidity of CHD patients after the first ACS. Furthermore, the effects of pharmacist care on adherence of evidence-based medications for CHD were also evaluated.

## Materials and Methods

### Design and Setting

This prospective, randomized trial was held from January 2016 to December 2016 in the cardiology ward of the Department of Cardiology at a university teaching hospital.

### Study Participants

Patients who suffered a first attack of ST-elevation myocardial infarction (STEMI), non-STEMI, or unstable angina with evidence of at least 50% occlusion of one or more major coronary arteries were considered for enrollment into the trial (Newby et al., 2006; Calvert et al., 2012).

Patients were excluded if they (1) had a history of percutaneous coronary intervention (PCI) before the current admission; (2) had a shorter hospital stay than needed for enrollment; (3) could not complete the expected 1-year follow-up for reasons such as severe heart failure, hepatic insufficiency, renal inadequacy, and respiratory failure or malignancy; (4) were cognitively impaired, had no self-care ability, had communication disorders, or were unable to participate in a follow-up telephone call; (5) were residents in a hospital or long-term care facility; (6) were allergic or contraindicated to the medicines used for secondary prevention of CHD; and (7) refused to participate. All participating patients provided informed consent.

### Randomization, Concealment, and Blinding

Randomization of the two groups was performed in a 1:1 ratio in blocks of 10 using a computer randomization sequence generation program after the patients signed their informed consent. The sequence was concealed until intervention was assigned. The unblinded researcher administered the randomization and contacted study pharmacists who then delivered the intervention to the included patients at discharge and in follow-ups after discharge. Pharmacists who were blind to the randomization status of participants recorded the baseline data from medical records and assessed the outcome data. The statistical analyses were conducted by the blinded researchers.

### Intervention and Control Components

Before discharge, the clinical pharmacists assessed whether patients in the intervention group were receiving the full complement of evidence-based medications for secondary prevention of CHD and, if not, whether there were documented contraindications for nonprescribed medications. If there were no contraindications for medications for secondary prevention of CHD, the pharmacists then contacted physicians with recommendations for secondary prevention medications. Patients in the intervention group received consultations by the clinical pharmacists four times: face to face at discharge (at the ward) and by telephone at 1 week and 1 and 3 months after discharge. At discharge, a printed discharge education sheet (**Supplemental Material Appendix A**) and consultations of medication therapy and lifestyle for secondary prevention of CHD were provided by the clinical pharmacists to each patient. A questionnaire was used to assess patients’ knowledge of self-management for CHD before and after the pharmacist-provided education at discharge. At the follow-up intervention after discharge, the clinical pharmacists assessed the drug-related problems of each patient by using a questionnaire (**Supplemental Material Appendix B**) and provided instructions on how to handle these drug-related problems. Patients in the control group received routine care performed by nurses, physicians, and dispensing pharmacists. Differences in activities between the intervention and control groups are shown in **Supplemental Material Appendix C**.

### Outcomes

All patients in both study arms were contacted by telephone at 6 and 12 months after hospital discharge by pharmacists who were blinded to the treatment groups. The primary outcomes of this study were the proportion of patients who had major adverse cardiovascular events (MACEs), including mortality, nonfatal myocardial infarction (MI), stroke, and unplanned cardiac-related rehospitalizations. Mortality included both cardiovascular and non-cardiovascular mortality. Secondary outcome was self-reported medication adherence to secondary prevention medications for CHD (antiplatelets, statins, β-blockers, and ACE inhibitors or ARBs). The pharmacists asked the patients whether they were using all of the medications as prescribed at discharge and whether there were any changes. In addition, if the patients picked up their refills at our outpatient pharmacy, the pharmacists would review the patients’ outpatient prescriptions to confirm the consistency.

### Sample Size

Since our study was the first study to use MACEs as a primary outcome to assess the pharmacist role in CHD patients, we did not know the exact percentage of reduction on MACEs resulting from the pharmacist intervention. We assumed that the rate of primary outcome would be 30% at the 12-month follow-up based on the previous trials (Mccollam and Etemad, 2005; Menzin et al., 2008; Chiang et al., 2014), and pharmacist intervention would lead to a relative risk reduction of 50%. To obtain 80% power and a two-sided α of 0.05 to detect a difference between groups, the trial would require 118 patients in each group (Chan, 2003). Additionally, total sample sizes of 200 patients were used in previous studies of pharmacist care among patients with heart failure (Stewart et al., 1999; Sadik et al., 2005). Based on these data, a sample size of 240 participants (120 per group) was selected for the present study.

### Statistical Analysis

We performed an intention-to-treat analysis that included all patients for primary outcome. In the secondary outcome analysis, patients who did not complete the 6- or 12-month follow-up assessment were excluded. Statistical analyses were conducted using the SPSS statistical software (version 20; SPSS Inc., Chicago, IL, USA). All baseline variables and outcomes were compared between the control and intervention groups using Wilcoxon rank sum tests for continuous variables and Pearson chi-square analysis or Fisher’s exact test for categorical variables. The means and standard deviations (SDs) are reported for continuous variables, and numbers and percentages are reported for all categorical variables. A two-sided *P* < 0.05 was considered statistically significant for all tests.

## Results

### Baseline Characteristics

**Figure 1:**
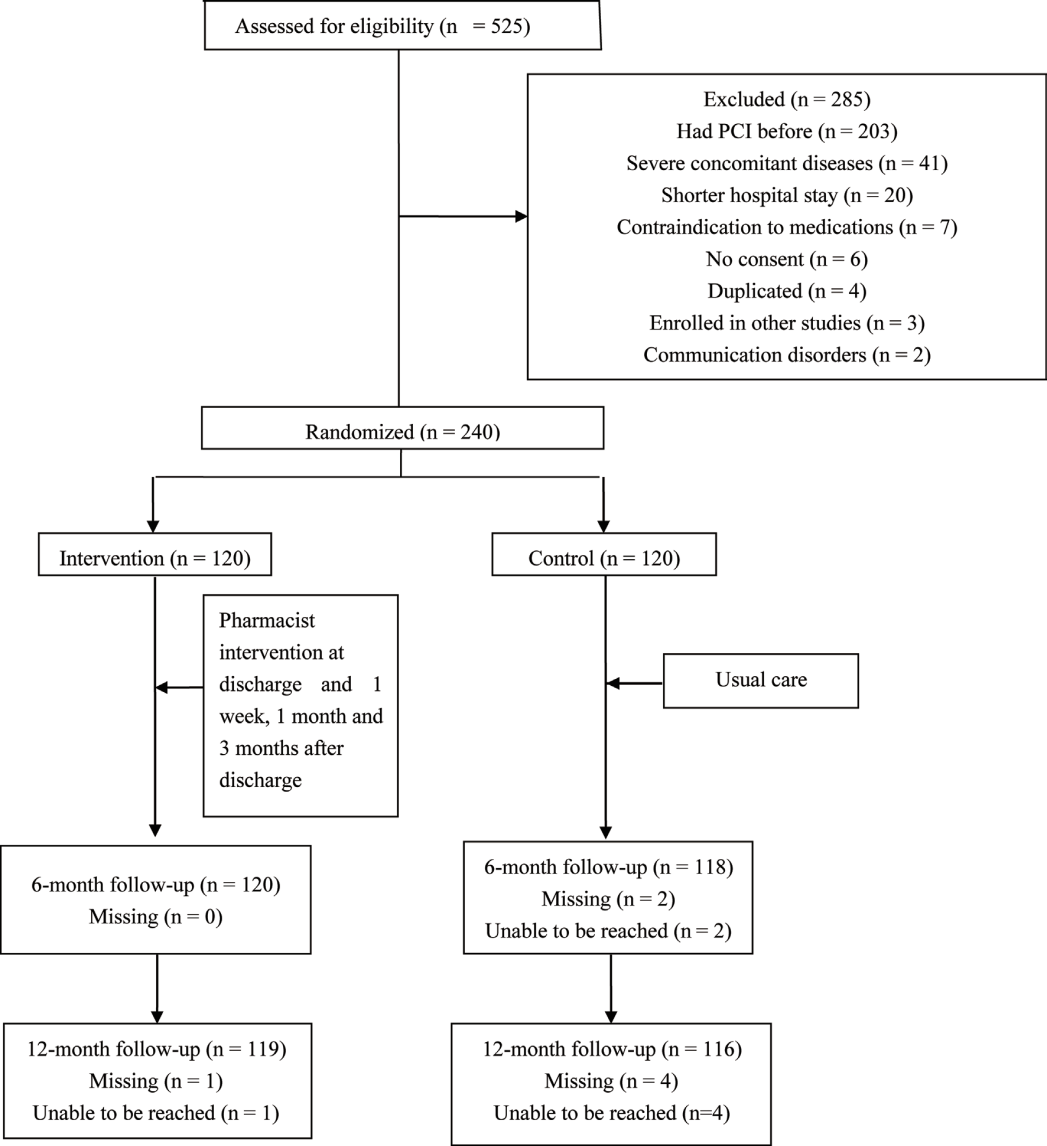
Trial protocol overview. PCI indicates percutaneous coronary intervention.

Of 525 patients screened, 240 subjects who met the inclusion criteria consented and were enrolled in this study (**Figure 1**). Two patients in the control group were lost at the 6-month follow-up (1.7%), and four patients in the control group (3.3%) and one patient in the intervention group (0.8%) were lost at the 12-month follow-up.

The mean age of the patients enrolled was 64 years, and 70.8% were male. There were no differences in characteristics, such as age, gender, medical insurance, body mass index (BMI), cardiovascular risk factors, lipid metabolisms profiles, renal functions, numbers of stenotic coronary arteries or stents applied, or left ventricular ejection fraction (LVEF) at baseline between the groups (**Table 1**). The proportions of the total patients who used antiplatelet drugs, statins, β-blockers, and ACE inhibitors/ARBs were 99.6%, 100%, 77.1%, and 78.5%, respectively. Significantly greater proportions of patients were prescribed a regimen of β-blockers and all four classes of evidence-based medications at discharge in the intervention group than in the control group (*P* = 0.001 and *P* = 0.009, respectively). Although a greater proportion of patients in the intervention group was prescribed ACE inhibitors/ARBs, this did not meet statistical significance (*P* = 0.091). There were no significant differences in the proportions of patients on a regimen of aspirin, P2Y_12_ receptor inhibitors, or statins between the two groups.

**Table 1:** Baseline and demographic characteristics of the study patients.

Characteristics	Intervention (*n* = 120)	Control (*n* = 120)	*P*
Age (years)	63.24 ± 10.19	64.12 ± 10.19	0.335
Male	81 (67.5)	89 (74.2)	0.256
Medical insurance	81 (67.5)	82 (68.3)	0.890
Height (cm)	164.89 ± 8.27	165.10 ± 11.03	0.856
Weight (kg)	65.61 ± 10.92	66.30 ± 11.04	0.769
BMI (kg/m^2^)	24.09 ± 2.96	24.36 ± 3.19	0.618
Education			0.475
College graduate	18 (15.0)	19 (15.8)	
High school graduate	21 (17.5)	16 (13.3)	
Middle school graduate	28 (23.3)	40 (33.3)	
Primary school graduate	41 (34.2)	34 (28.3)	
Illiterate	12 (10.0)	11 (9.2)	
Cardiovascular risk factor			
Hypertension	82 (68.3)	81 (67.5)	0.890
Hyperlipidemia	46 (38.3)	40 (33.3)	0.419
Obesity	49 (40.8)	50 (41.7)	0.896
Diabetes mellitus	35 (29.4)	39 (32.5)	0.606
Current smoker	49 (40.8)	42 (35.0)	0.352
Last BP recorded in medical records (mmHg)			
SBP	132.47 ± 21.74	133.05 ± 17.71	0.826
DBP	76.24 ± 12.47	75.59 ± 11.34	0.576
Heart rate	78.14 ± 12.37	75.45 ± 11.44	0.142
Last cholesterol recorded in medical records (mmol/L)			
TC	4.39 ± 1.12	4.25 ± 1.08	0.440
LDL-C	2.38 ± 0.85	2.28 ± 0.75	0.465
Creatinine (mg/ml)	72.22 ± 18.84	71.89 ± 17.11	0.790
Fasting blood glucose (mmol/L)	5.74 ± 1.50	5.76 ± 1.29	0.508
Diagnosis of ACS			0.114
ST-elevation MI	31 (25.8)	25 (20.8)	
Non-ST-elevation MI	17 (14.2)	9 (7.5)	
Unstable angina	72 (60.0)	86 (71.7)	
No. of stenotic coronary arteries			0.739
1	61 (50.8)	59 (49.2)	
2	32 (26.7)	29 (24.2)	
3	27 (22.5)	32 (26.7)	
No. of stents during the hospitalization			0.212
0	35 (29.2)	37 (30.8)	
1	64 (53.3)	55 (45.8)	
2	18 (15.0)	18 (15.0)	
≥3	3 (2.5)	10 (8.3)	
Thrombus aspiration	16 (13.3)	10 (8.3)	0.213
LVEF, %	63.11 ± 9.23	63.07 ± 10.27	0.568
Medication prescribed at discharge			
Antiplatelet	120 (100)	119 (99.2)	1.000
Aspirin	112 (93.3)	108 (90.0)	0.350
P2Y_12_ receptor inhibitor	105 (87.5)	109 (90.8)	0.406
Statin	120 (100)	120 (100)	n/a
ACE inhibitor/ARB	98 (81.7)	87 (72.5)	0.091
β-Blocker	105 (87.5)	84 (70.0)	0.001
All 4 classes	89 (74.2)	70 (58.3)	0.009

Categorical variables are presented as number (%), and continuous variables are presented as the mean ± SD. Obesity was defined as BMI ≥ 25 kg/m^2^. BMI, body mass index; BP, blood pressure; SBP, systolic blood pressure; DBP, diastolic blood pressure; TC, total cholesterol; LDL-C, low-density lipoprotein cholesterol; ACS, acute coronary syndrome; MI, myocardial infarction; LVEF, left ventricular ejection fraction; ACE, angiotensin-converting enzyme; ARB, angiotensin receptor blocker.

### Primary Endpoints

Among 240 participants analyzed, the percentage of total MACEs was 5.5% at the 6-month follow-up and 11.5% at the 12-month follow-up (**Table 2**). At 6 months, the proportion of total MACEs was 3.3% in the intensive follow-up group and 7.6% in the control follow-up group, while at 12 months, the proportions of total MACEs were 10.9% and 12.1%, separately. There were no significant differences in the percentages of total MACEs, all-cause death, MI, stroke, or cardiac-related rehospitalization at the 6- or 12-month follow-up between the two groups. There were fewer rehospitalizations in the intervention group than in the control group at the 6-month follow-up, although this difference did not meet statistical significance (*P* = 0.071).

**Table 2:** Comparison of primary and secondary endpoints in the two groups (within 6 and 12 months after discharge).

	6 months			12 months		
	Intervention	Control	*P*	Intervention	Control	*P*
Primary endpoints						
Total MACEs	4 (3.3)	9 (7.5)	0.154	13 (10.8)	14 (11.7)	0.838
All-cause death	0	0	n/a	0	2 (1.7)	0.498
MI	0	1 (0.8)	1	0	1 (0.8)	1
Stroke	1 (0.8)	0	1	2 (1.7)	0	0.498
Rehospitalization	3 (2.5)	9 (7.5)	0.076	11 (9.2)	12 (10.0)	0.826
Secondary endpoints						
Medication adherence						
Antiplatelet	114 (95.0)	111 (94.1)	0.783	111 (93.3)	106 (91.4)	0.631
Aspirin	98 (81.7)	96 (81.4)	0.951	98 (82.4)	91(78.4)	0.451
P2Y_12_ receptor inhibitor	100 (83.3)	99 (83.9)	0.906	86 (72.3)	87 (75.0)	0.635
Statin	111 (92.5)	109 (92.4)	0.970	110 (92.4)	105 (90.5)	0.598
ACE inhibitor/ARB	67 (55.8)	71 (60.2)	0.498	70 (58.8)	68 (58.6)	0.975
β-Blocker	89 (74.2)	76 (64.4)	0.103	89 (74.8)	74 (63.8)	0.068
All 4 classes	58 (48.3)	54 (45.8)	0.691	57 (47.9)	54 (46.6)	0.836

MACEs: major adverse cardiac events, including mortality, nonfatal MI, stroke, and cardiac-related rehospitalization; MI, myocardial infarction; ACE, angiotensin-converting enzyme; ARB, angiotensin receptor blocker.

### Secondary Endpoints

The self-reported proportions of the total patients who adhered to antiplatelet drugs, statins, β-blockers, and ACE inhibitors/ARBs were 94.5%, 92.4%, 69.3%, and 58.0% at 6 months and 92.3%, 91.5%, 69.4%, and 58.7% at 12 months, respectively. Although the proportion of those taking all four classes of medications at discharge was higher in the intervention group than in the control group (74.2% and 58.3%, respectively; *P* = 0.009), there was no difference at 6 and 12 months, respectively (*P* = 0.691 and *P* = 0.836). Furthermore, there were no differences between the intervention and control groups in the proportions of patients who adhered to antiplatelet drugs, statins, and ACE inhibitor/ARBs at the 6- and 12-month follow-ups (**Table 2**). A non-statistically higher proportion of patients was adherent to β-blockers in the intervention group both at the 6-month follow-up (*P* = 0.103) and at the 12-month follow-up (*P* = 0.068).

## Discussion

In this randomized controlled study, we found that pharmacist intervention did not significantly reduce the cardiovascular events in patients with CHD within 1 year. Pharmacist interventions increased β-blockers and ACE inhibitors/ARBs use at discharge but did not improve medication adherence at 6 and 12 months after discharge. Although the results were negative, our findings have several implications for the future research design of pharmacist intervention on patients with CHD.

Our study used “hard endpoints” (such as mortality, cardiovascular events, and hospitalizations) as primary outcomes, which is unique to our knowledge in studies evaluating the pharmacist role in the management of CHD (Cai et al., 2013). A prior study conducted by Ho et al. (2014), which used clinical endpoints (mortality, MI, and revascularization) as a secondary outcome for evaluating the effect of pharmacist intervention on patients with ACS, also had negative clinical outcome results. Several reasons are possible in interpreting why the MACEs were nonsignificantly decreased in the intervention group compared with the control group in this trial. First, because the rate of the primary outcome was lower than expected, the sample size did not have enough statistical power to detect a significant difference between the two groups. We assumed that the rate of primary outcome would be 30% at the 12-month follow-up based on the previous studies (Mccollam and Etemad, 2005; Menzin et al., 2008; Chiang et al., 2014), but actually the MACEs rate was 11.5%. Although there was not adequate statistical power to detect a significant difference between the two groups, a trend toward a relative risk reduction by more than 50% on total MACEs and on rehospitalization in the intervention group at the 6-month follow-up should encourage further investigation of the impact of pharmacist intervention on the mortality and morbidity of CHD patients. Additionally, we noticed that this trend toward fewer cardiovascular events was not apparent at the 12-month follow-up. In our study, pharmacist intervention mainly focused on the first 3 months after discharge. We supposed that early intensive intervention performed by clinical pharmacists could change the behavior of CHD patients and maintain these positive effects until the end of follow-up. However, changing the behavior of patients may be a long-term process, and more interventions need to be carried out by clinical pharmacists after 6 months when patients had been discharged from the hospital. Further studies with larger sample sizes and longer time frames for interventions are needed to evaluate the effects of clinical pharmacist intervention in patients with CHD on clinical outcome events. Second, it may take longer than the 1-year study period to see changes. Du et al. (2016) study showed that the MACE rate was markedly decreased at 36 months by a cardiologist-coordinated intensive follow-up program compared with the usual follow-up, but there was no significant difference between the two groups at 12 months. In future studies, extended follow-up time is needed to assess the effect of pharmacist intervention. Third, the telephone call follow-up at 6 months in both intervention and control groups may have an impact on endpoints at 1 year.

Although medications such as aspirin, statins, β-blockers, and ACE inhibitors/ARBs have been shown to reduce morbidity and mortality in patients with ACS, these agents are not optimally used (Bailey et al., 2007; Bagnall et al., 2010). Our previous study showed that in Chinese hospitals, the use of antiplatelet agents and statins was optimal, but the use of β-blockers and ACE inhibitors/ARBs was underutilized at discharge in patients with CHD (Xu et al., 2014). In this study, clinical pharmacists significantly improved the prescription of four classes of evidence-based medications for CHD at discharge, especially in the use of β-blockers. However, our results did not show an increase in the proportion of patients taking evidence-based medications at 6 and 12 months after discharge by the pharmacist intervention. Previous studies conducted by Calvert et al. (2012) and Schwalm et al. (2015) also found that pharmacist intervention failed to improve self-reported adherence to cardiovascular medications. We speculated this may be because patients tend to overreport their medication adherence. In the study conducted by Calvert et al. (2012), although self-reported adherence to β-blockers was not different between the intervention and control arms, the adherence to β-blockers using prescription refill records was much lower than self-reported and was statistically significantly better in intervention versus control at 6 months after discharge. Therefore, medication adherence measured by using pharmacy refill data may be more sensitive to detect an effect of intervention. Unfortunately, the data of drug use among different hospitals in China have not been shared online; we could not get the prescription refill records of the included patients from other hospitals online, so we chose self-reported adherence in this study. Our study showed a nonsignificant improvement in patient’s adherence to β-blockers after hospital discharge, which is also very promising as a direction for pharmacist intervention. According to a recent review, it is unlikely that medication adherence and cardiovascular outcomes can be improved in subjects who are already highly adherent (Doggrell, 2019). Since there is high adherence to antiplatelets and statins in patients with CHD, which was also observed from our study, more effort on improving medication adherence to β-blockers and ACE inhibitor/ARBs should be strengthened in future studies.

This study raises questions about the effectiveness of pharmacist intervention in the management of CHD. While some studies have shown positive effects of pharmacist intervention on medication adherence and lipid management in CHD patients (Faulkner et al., 2000; Straka et al., 2005; Ho et al., 2014; Nguyen et al., 2018), there is heterogeneity in the results (Community Pharmacy Medicines Management Project Evaluation Team, 2007; Calvert et al., 2012; Schwalm et al., 2015). So far, the number of studies evaluating the contribution from pharmacists in the management of CHD is small, and most of these studies included a limited number of participants; it is necessary to conduct further studies to confirm the role of pharmacists in the secondary prevention of CHD.

This study has several limitations. First, this study is a single-center study, which was conducted at an academic medical center with a low number of participants. As mentioned above, the small sample size used in this study may result in a lower power to detect differences between the study arms. Second, medication adherence was reported by the patient, which may overestimate the adherence. To reduce this bias, before calling the patient, the follow-up pharmacists searched the electronic medical record system to determine whether the patient had subsequent visits in our hospital during the follow-up period. For those patients who had subsequent visits in our hospital, their prescribed medications would be recorded by the follow-up pharmacist and verified with the patients. Despite these limitations, our results add to the existing literature about the effectiveness of disease management by pharmacist for patients with CHD.

In summary, the clinical pharmacist intervention did not improve mortality and morbidity of CHD patients overall. This study adds to the existing literature by leaving open questions about the effectiveness of clinical pharmacist intervention on the mortality and morbidity of CHD patients. Further studies with larger sample sizes and longer time frames for both intervention and follow-up are needed to validate the role of clinical pharmacists in the morbidity and mortality of CHD.

## Data Availability Statement

The raw data supporting the conclusions of this manuscript will be made available by the authors, without undue reservation, to any qualified researcher.

## Ethics Statement

The studies involving human participants were reviewed and approved by Institute Ethics Committee of Second Affiliated Hospital, Zhejiang University School of Medicine. The patients/participants provided their written informed consent to participate in this study. Written informed consent was obtained from the individual(s) for the publication of any potentially identifiable images or data included in this article.

## Author Contributions

HX, HD, and XYan designed the research. HX and HD conducted the literature search and analyses and wrote the first draft of the paper. JZ, XYe, JH, LG, SL, JW, and CH contributed to acquisition of data. All authors interpreted the data, revised the subsequent drafts for important intellectual content, and read and approved the final manuscript. HD takes responsibility for the integrity of the data and the accuracy of the data analysis.

## Funding

This study was supported by research grants from the National Natural Science Foundation of China (81703479 and 81773700), the Health Bureau of Zhejiang Province (WKJ-ZJ-1719), and the Special Research Fund of Jiangsu Chia-Tai Tianqing Pharmacy. The funders had no role in the study design or conduct, data collection and analysis, decision to publish, or the preparation or approval of the manuscript.

## Conflict of Interest

The authors declare that the research was conducted in the absence of any commercial or financial relationships that could be construed as a potential conflict of interest.

## Abbreviations

CHD, coronary heart disease; ACS, acute coronary syndrome; ACE, angiotensin-converting enzyme; ARBs, angiotensin receptor blockers; CVDs, cardiovascular diseases; SAHZU, the Second Affiliated Hospital, Zhejiang University School of Medicine; STEMI, ST-elevation myocardial infarction; PCI, percutaneous coronary intervention; MACEs, major adverse cardiovascular events; MI, myocardial infarction; SDs, standard deviations; BMI, body mass index; BP, blood pressure; SBP, systolic blood pressure; DBP, diastolic blood pressure; LVEF, left ventricular ejection fraction; TC, total cholesterol; LDL-C, low-density lipoprotein cholesterol.
