# Supplementary material for: Impacts of Clinical Pharmacist Intervention on the Secondary Prevention of Coronary Heart Disease: A Randomized Controlled Clinical Study
Source: Front Pharmacol. 2019 Oct 8;10:1112. doi: 10.3389/fphar.2019.01112 (PMC6791923; doi:10.3389/fphar.2019.01112)
Supplement: Supplementary file 1 [file DataSheet_1.docx]

**SUPPLEMENTAL MATERIAL**

**Appendix A. Main contents of the printed discharge education sheet for prevention of coronary heart disease (CHD) produced by the research pharmacist**

What is CHD

Symptoms of CHD

What is secondary prevention for patients with CHD

Risk factor (smoking, blood pressure, lipid, physical activity, weight and diabetes) management goals for CHD

Lifestyle change recommendations for CHD: stop smoking, choose a healthy diet, maintain a healthy weight, be physically active, have an influenza vaccination.

Medications for secondary prevention of CHD: effects, duration, potential adverse effects and precautions for use of antiplatelets, statins, β-blockers, and angiotensin-converting enzyme (ACE) inhibitors or angiotensin receptor blockers (ARBs)

**Appendix B. Questionnaire used to identify drug-related problems for telephone intervention at 1 week, 1 month and 3 months after discharge**

Is the patient currently taking medications that were prescribed at discharge?

Whether any medication changes have been made

Whether missed medication doses

If the patient is not taking medications prescribed, what is the rationale?

Whether have had any recurrence of chest pain

Whether have had any adverse drug effects, such as bleeding, hypotension, bradycardia, dry cough, myalgia, upset stomach, etc.

Whether have any questions or concerns about their medication use

Assess the blood pressure, heart rate, weight and laboratory results of the patient at 1 month and 3 months after discharge

Pharmacist’s instructions

**Appendix C. Comparison of activities in usual care versus intervention arms**

|  | Usual care | Intervention |
| --- | --- | --- |
| Written patient informed consent | × | × |
| Collecting baseline characteristics of patients | × | × |
| Routine discharge counseling from patient-care physician, nurse or delivery pharmacist | × | × |
| Additional counseling from clinical pharmacist on the risk factor control, importance of medication adherence and the effects and potential adverse effects of medications for CHD at discharge |  | × |
| Assessing patients’ understanding of self-management for CHD |  | × |
| Enhanced follow-up 1 week, 1 and 3 months after discharge by telephone from clinical pharmacist to assess the risk factor control, medication adherence, adverse effects of medications |  | × |
| Follow-up 6 and 12 months after discharge by telephone from blinded pharmacist to assess the risk factor control, medication adherence, adverse effects of medications, and MACEs | × | × |

CHD indicates coronary heart disease; MACEs, major adverse cardiac events, including mortality, non-fatal myocardial infarction (MI), stroke and unplanned cardiac-related re-hospital admissions.
